# Insights into the Superoxide Dismutase Gene Family and Its Roles in *Dendrobium catenatum* under Abiotic Stresses

**DOI:** 10.3390/plants9111452

**Published:** 2020-10-28

**Authors:** Hui Huang, Hui Wang, Yan Tong, Yuhua Wang

**Affiliations:** 1 Yunnan Key Laboratory for Wild Plant Resources, Kunming Institute of Botany, Chinese Academy of Sciences, Kunming 650201, China; huanghui@mail.kib.ac.cn (H.H.); wanghui1@mail.kib.ac.cn (H.W.); tongyan@mail.kib.ac.cn (Y.T.); 2 University of Chinese Academy of Sciences, Beijing 100049, China

**Keywords:** *Dendrobium catenatum*, superoxide dismutase (SOD), gene family, gene expression, stresses

## Abstract

*Dendrobium catenatum* is a member of epiphytic orchids with extensive range of pharmacological properties and ornamental values. Superoxide dismutase (SOD), a key member of antioxidant system, plays a vital role in protecting plants against oxidative damage caused by various biotic and abiotic stresses. So far, little is known about the SOD gene family in *D. catenatum*. In this study, eight SOD genes, including four Cu/ZnSODs, three FeSODs and one MnSOD, were identified in *D. catenatum* genome. Phylogenetic analyses of SOD proteins in *D. catenatum* and several other species revealed that these SOD proteins can be assigned to three subfamilies based on their metal co-factors. Moreover, the similarities in conserved motifs and gene structures in the same subfamily corroborated their classification and inferred evolutionary relationships. There were many hormone and stress response elements in *DcaSODs*, of which light responsiveness elements was the largest group. All *DcaSODs* displayed tissue-specific expression patterns and exhibited abundant expression levels in flower and leaf. According to public RNA-seq data and qRT-PCR analysis showed that the almost *DcaSODs*, except for *DcaFSD2*, were highly expressed under cold and drought treatments. Under heat, light, and salt stresses, *DcaCSD1*, *DcaCSD2*, *DcaCSD3* were always significantly up-regulated, which may play a vital role in coping with various stresses. The expression levels of *DcaFSD1* and *DcaFSD2* were promoted by high light, suggesting their important roles in light response. These findings provided valuable information for further research on *DcaSODs* in *D. catenatum*.

## 1. Introduction

*Dendrobium catenatum*, belonging to *Dendrobium* genus (Orchidaceae), is a member of epiphytic orchids which takes root on the surface of tree bark or rocks [1]. Due to the special living environment, *D. catenatum* evolved novel features and sophisticated defense mechanisms that allow it to exploit its environment and against serious abiotic stresses, including thick leaves, abundant polysaccharides and facultative crassulaceaen acid (CAM) metabolism that is a photosynthetic pathway with high water-use efficiency [2,3,4,5,6]. *D. catenatum* is considered to be drought-resistant material useful for elucidating mechanisms of mitigating drought stress [2,5,6]. Additionally, *D. catenatum* is a well-known traditional Chinese medicinal herb, and has both an extensive range of pharmacological properties and ornamental values. Stem of *D. catenatum* contains a large number of polysaccharides that exhibits anti-inflammatory, immune-enhancing, antioxidant, and anti-glycation activities [7,8]. Light and water affected significantly the accumulation of polysaccharides [2,6]. Consequently, study on the *D. catenatum* not only has important scientific value, but also has important economic value.

The increased production of toxic reactive oxygen species (ROS) is considered to be a universal or common feature of stress conditions. ROS includes hydrogen peroxide (H_2_O_2_), superoxide anion radicals (O_2_^.-^), peroxyl radicals (HOO-), hydroxyl radicals (OH-), and singlet oxygens (^1^O_2_) [9,10]. The antioxidant defense system constitutes the first line of defense against ROS produced in response to abiotic stresses. The superoxide dismutase (SOD) can catalyze the dismutation of supeoxide to H_2_O_2_ and O_2_^.^ and is one of the most effective components of the antioxidant defense system in plant cells against ROS toxicity. SODs, which are localized at different cellular compartments, could be categorized into three subgroups based on metal co-factors: Cu/ZnSOD, FeSOD and MnSOD [11]. Because of their crucial roles in the antioxidant system, SODs have been reported to be involved in protection against abiotic stresses such as heat, cold, drought and salinity [12,13,14,15]. For example, Overexpression of Cu/ZnSODs in potato indicated that transgenic plants exhibited increased tolerance to oxidative stress [12]. Asensio et al. [14] indicated that stress conditions, such as nitrate excess or drought markedly increased anti-cytosolic FeSOD (cyt-FeSOD) contents in soybean tissues. Currently, the SOD gene family had been identified in many species, such as banana [15], *Arabidopsis thaliana* [16], tomato [17], cucumber [18], cotton [19], wheat [20], and *Salvia miltiorrhiza* [21]. To date, the characters of *SOD* genes and their roles in stress resistance of *D. catenatum* are still largely unknown. In this study, we perform a genome-wide analysis of *SOD* genes in *D. catenatum* genome, and investigated their characteristics, including physicochemical properties, structural characteristics and evolutionary relationships, and responses to abiotic stress. Together, our study provides a foundation for further investigation into its function of the SOD family in *D. catenatum*.

## 2. Results

### 2.1. Identification of SOD Gene Family Members in Dendrobium catenatum

After strict screening, a total of eight *DcaSOD* genes were identified in the *D. catenatum* genome (Table 1, Table S1). These *DcaSOD* genes were termed as *DcaCSD1*, *DcaCSD2*, *DcaCSD3*, *DcaCSD4*, *DcaFSD1*, *DcaFSD2*, *DcaFSD3* and *DcaMSD1*, and were assigned to three subfamilies according to their functional annotations. The number of amino acids of DcaSOD proteins ranged from 269 amino acids (aa) (DcaFSD2) to 76 aa (DcaFSD3) with an average of 196 aa. The molecular weights (MW) changed from 15.31 (DcaCSD2) to 30.78 kDa (DcaFSD2) with isoelectric points of 4.91 (DcaFSD2)—8.61 (DcaMSD1). The subcellular localization of eight DcaSOD proteins were predicted by ProComp. The results showed that DcaCSD1, DcaFSD1, and DcaFSD2 may be located in the chloroplast. DcaCSD2, DcaCSD3 and DcaCSD4 were predictably located in the cytoplasmic. In addition, the DcaMSD1 was predictably located in the mitochondria. The grand average of hydropathicity (GRAVY) of these DcaSOD proteins implied that DcaCSD1 and DcaFSD3 were hydrophobic protein, and other DcaSOD proteins were hydrophilic protein.

### 2.2. Phylogenetic Analysis of DcaSOD Proteins

**Figure 1:**
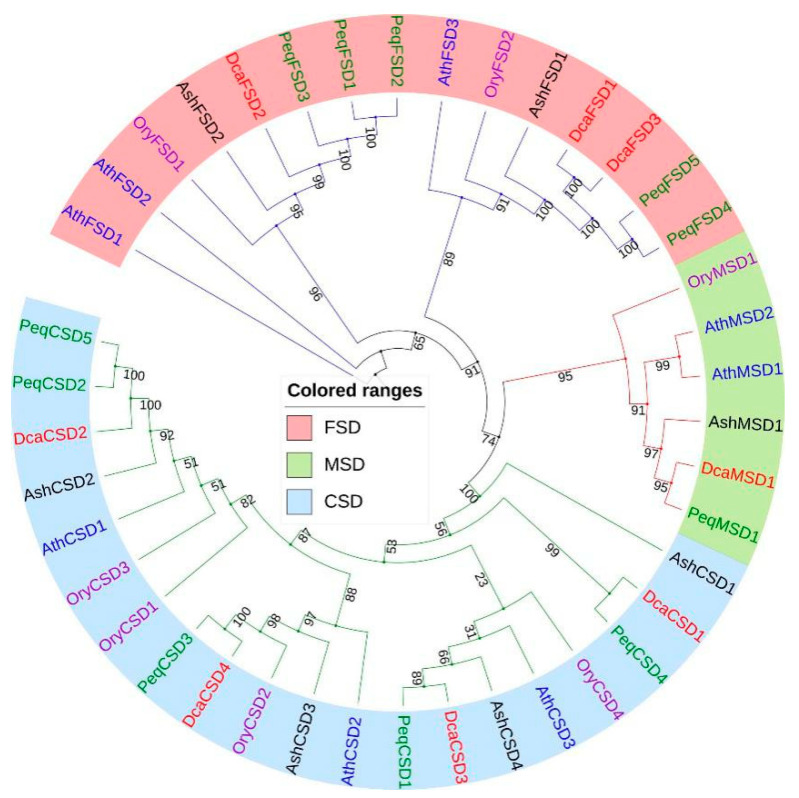
Phylogenetic tree of SOD proteins from *D. catenatum, Arabidopsis,* *Oryza sativa*, *Phalaenopsis equestris* and *Apostasia shenzhenica*. The phylogenetic tree was constructed using the Maximum-Likelihood (ML) method with 1000 bootstrap replications. The three subfamilies were distinguished in different colors. The identified DcaSOD proteins were highlighted by red.

To further explore the classification and evolutionary characteristics of these DcaSODs, multiple sequence alignment of DcaSOD protein sequences with their homologs from *Arabidopsis*, *Oryza sativa*, *Phalaenopsis equestris* and *Apostasia shenzhenica* was carried out. An un-rooted phylogenetic tree showed that all *SOD* genes were divided into three groups, named CSD, FSD and MSD subfamily (Figure 1, Table S1). The CSD subfamily had the most members (20), followed by the FSD (15) and MSD subfamily (6). We found that all DcaSOD proteins were much closer to PeqSOD proteins than AshSOD proteins.

### 2.3. Conserved Motifs, Gene Structures, Distribution, and cis-Elements Analysis

**Figure 2:**
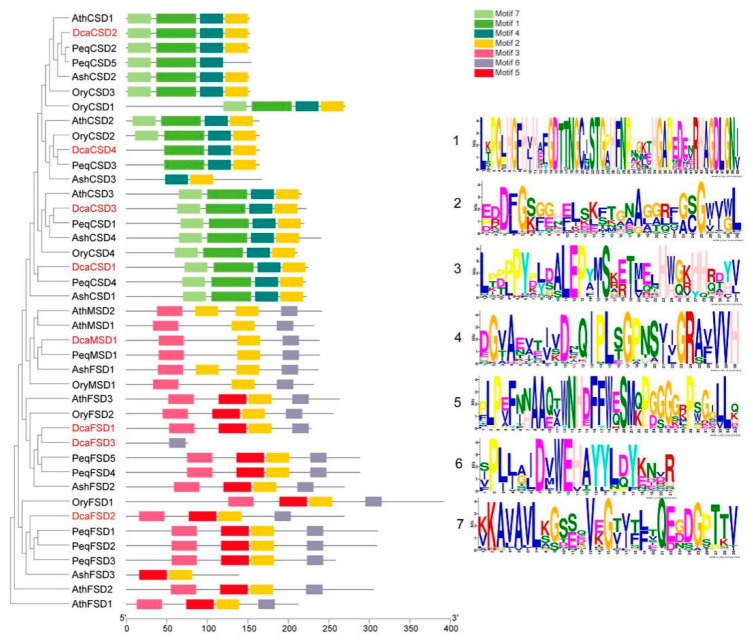
The motif composition and distribution of SOD proteins in *D. catenatum, Arabidopsis,* *O. sativa* and *P. equestris* and *A. shenzhenica*. The colored boxes with numbers represent seven motif. The identified DcaSOD proteins were highlighted by red.

**Figure 3:**
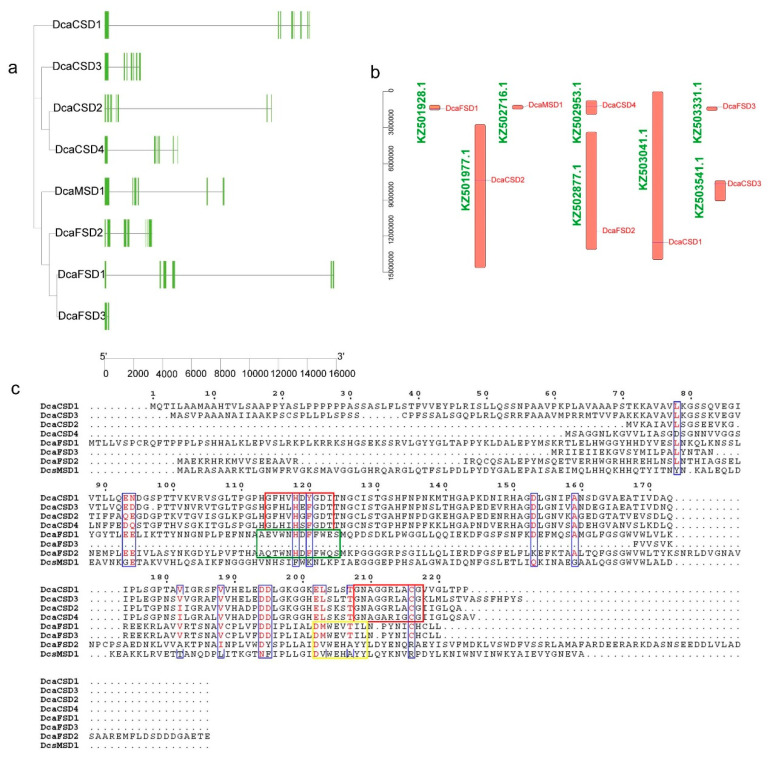
Gene structures, locations, and multiple sequence alignment of *DcaSOD* genes. (**a**) Gene structures of *DcaSOD* genes. Exons and introns were represented by green ellipses and gray lines, respectively. (**b**) Locations of *DcaSOD* genes. The orange bars indicate the different scaffolds of *D. catenatum* genome. (**c**) Multiple sequence alignment of amino acid sequences of DcaSOD proteins. Cu/ZnSOD signatures (G[FL]H[VLI]H[DEGS][FY]GD[TI]) and (GNAG[GA]R[LI][AG]CG) are boxed in red. The conserved metal-binding domain (D[MV]WE[VH][TA][IY][LY]) for Fe-MnSOD is in yellow box. The signature (A[EQ][VT]WNHDFFW[EQ]S) responsible for the recognition of iron ion by FeSODs in boxed in green.

A total of seven motifs ranging from 21 to 50 aa were searched by MEME analysis. As shown in Figure 2, almost all members in the same subfamily shared common motif compositions with each other, suggesting functional similarities among these SOD proteins within the same subfamily. Motif 2 was widely distributed across almost all SOD proteins, except for PeqCSD5 and DcaFSD3. The members of CSD subfamily contained the motifs 1, 2, 4, and 7. The members of MSD subfamily contained the motifs 2, 3, and 6. Motif 5 was presented in the members of FSD subfamily, except for DcaFSD3. The predicted exon-intron structures were analyzed to gain an insight into the variation of *SOD* genes in *D. catenatum*. The results showed that the exons numbers of *DcaSOD* genes ranged from 2 (*DcaFSD3*) to 8 (*DcaCSD1*) (Figure 3a). In addition, the identified eight *DcaSOD* genes were mapped onto scaffolds, which showed that they distributed in eight different scaffolds (Figure 3b). Multiple sequence alignment of the eight DcaSOD amino acid sequences was performed and the results showed that all DcaCSDs contained the Cu/ZnSOD signatures (G[FL]H[VLI]H[DEGS][FY]GD[TI]) and (GNAG[GA]R[LI][AG]CG) (Figure 3c). The conserved metal-binding domain (D[MV]WE[VH][TA][IY][LY]) were found in DcaFSDs and DcaMSD. In addition, the signature (A[EQ][VT]WNHDFFW[EQ]S) responsible for the recognition of iron ion by FeSODs were identified in DcaFSDs, except for DcaFSD3.

**Figure 4:**
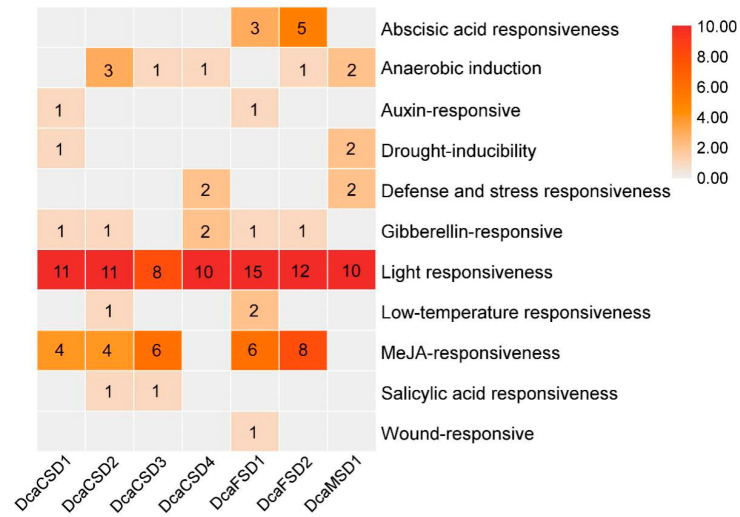
*Cis*-elements in promoters of *DcaSOD* genes that are related to hormone and stresses responses. The bar indicates that the number of *cis*-elements.

To further explore the potential functions of *DcaSOD* genes during plant growth and stress responses, the sequences of the 2.0 kb region upstream of the translation initiation site of each of *DcaSOD* genes were analysis using the PlantCARE. In total, 142 *cis*-elements related to hormones and stresses responses were identified in all identified *DcaSOD* promoters (Figure 4). Among these predicted *cis*-elements, the light responsiveness element was the largest group including Box 4 (25), G-Box (7), GT1-motif (10), I-box (8), TCT-motif (8), TCCC-motif (2) and others (17) (Table S2). The Box 4 (25), the most light-responsive element was present in 6 *DcaSOD* promoters. Fourteen CGTCA-motif and 14 TGACG-motif, both of which are involved in MeJA-responsiveness, were identified in five *DcaSOD* promoters, respectively. ABRE was abscisic acid (ABA) responsiveness element and was present exclusively in *DcaFSD1 and DcaFSD2* promoters. *DcaCSD2* and *DcaFSD1* promoters contained low-temperature-responsive element (LTR) that was involved in response to cold stress. TATC-box and P-box that were gibberellin-responsive element were found to be present in three and three *DcaSOD* promoters, respectively. The TCA-element that was involved in salicylic acid responsiveness were found to be present in *DcaCSD2* and *DcaCSD3* promoters. In addition, eight ARE *cis*-elements involved in anaerobic induction were identified in five *DcaSOD* promoters. There was only a *cis*-element was identified in *DcaFSD3* promoter, which is due to many gaps in its promoter region.

### 2.4. Distinct Expression Profiles of DcaSOD Genes in Different Tissues, Cold and Drought Responses

**Figure 5:**
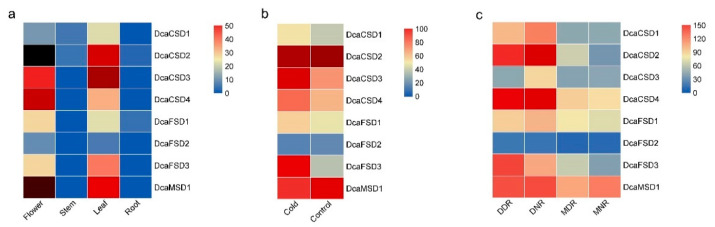
Expression profiles of *DcaSOD* genes in different tissues, cold and drought treatments. (**a**) Tissues. (**b**) Cold treatment. (**c**) Drought treatment. The Fragments Per Kilobase Million (FPKM) values of genes in samples were showed by different colored rectangles. Red indicates high expression level. Blue indicates low expression level.

To analyze the expression profiles of eight *DcaSOD* genes, we investigated their transcripts abundance patterns across multiple tissues including flower, leaf, stem, and root based on public RNA-seq data. The heat map showed that almost all *DcaSOD* genes had tissue-specific expression patterns (Figure 5a). Most of them were highly expression level in flower and leaf, especially *DcaCSD2*, *DcaCSD3*, *DcaCSD4* and *DcaMSD1,* and were lowly expressed in root and stem. Under cold treatment (0 °C for 20 h), the expression levels of *DcaCSD1*, *DcaCSD3*, *DcaCSD4*, *DcaFSD1* and *DcaFSD3* increased by cold treatment (Figure 5b). Of them, the expression level of *DcaFSD3* under cold treatment was more than three times than that of control. *DcaCSD2* and *DcaMSD1* showed constitutive expression with high expression levels in leaves under control and cold treatment.

To achieve a better understanding of the roles of SODs under drought in *D. catenatum,* we used public RNA-seq data to analyze the transcriptomes of leaves under control (30–35% volumetric water content) and serious drought (0% volumetric water content), were collected at 09:00 (designed as moist-day MDR and dried-day DDR) and 21:00 (designed as moist-night MNR and dried-night DNR) (Figure 5c). Under drought treatment, the expression of all *DcaSOD* genes were up-regulated at night and in the daytime. The expression levels of *DcaCSD2* were six and two times under drought than that of control at night and in the daytime, respectively. The expression levels of *DcaCSD1* were three and two times under drought than that of control at night and in the daytime, respectively. It is notable that the four *DcaCSDs* were highly expressed in DNR than in DDR. Among the FSD subfamily, the expression levels of *DcaFSD3* were three and two times under drought than that of control at night and in the daytime, respectively. After differentially expressed genes (DEGs) identification between DDR, DNR, MDR and MNR, a total of 5478 DEGs were identified (fold change ≥ 2 and FDR ≤ 0.01) (Table S3). *DcaCSD1* and *DcaCSD2* genes were annotated as DEGs. The Pearson correlation analysis was carried out to predict the relationship between the two *DcaSODs* and other DEGs, considering positive (≥0.99) and negative (≤−0.99) relationship with *p*-value < 0.005 (Table 2). In our result, two genes showed negative correlation with the *DcaCSD1*, while 13 genes were detected as positive. There were 12 negative correlation genes and three positive correlation genes with the *DcaCSD2*.

### 2.5. qRT-PCR Verified the Expression of DcaSOD Genes in Response to Heat, Light and NaCl Treatments

**Figure 6:**
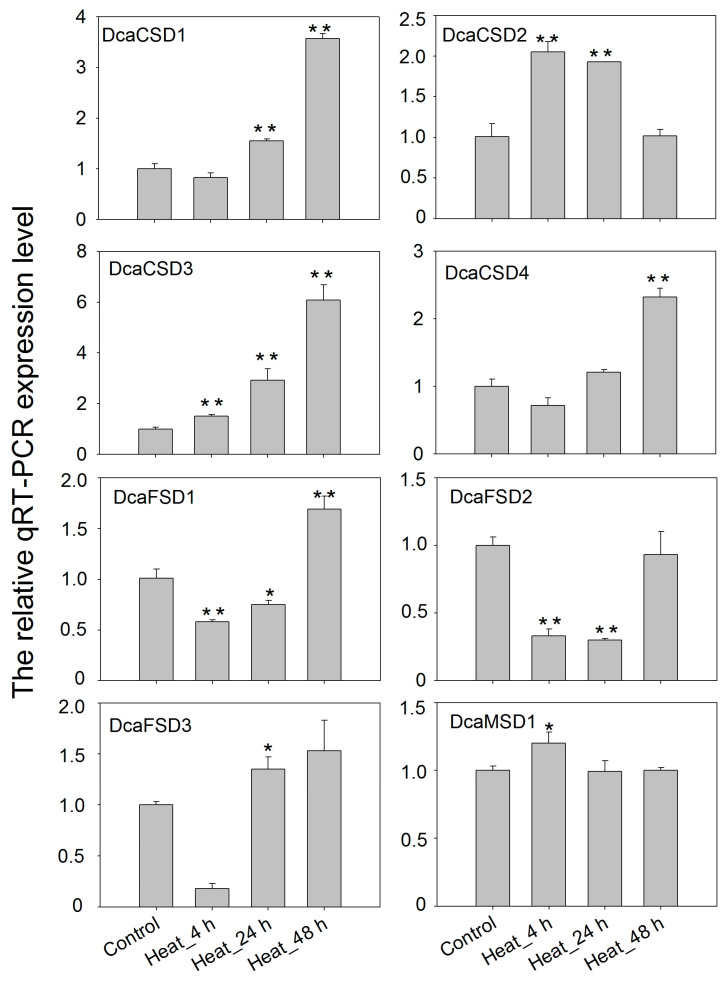
qTR-PCR analysis of the expression patterns of *DcaSOD* genes under heat treatments. The relative qRT-PCR expression levels were calculated with 2^−^^∆∆CT^ and the *DcaActin* gene was used as endogenous reference gene. Error bars represent the standard deviation of three replications. Bars marked with asterisks indicate significant differences (Student’s *t*-test) to corresponding control samples for the time points under treatments (* *p* < 0.05, ** *p* < 0.01).

**Figure 7:**
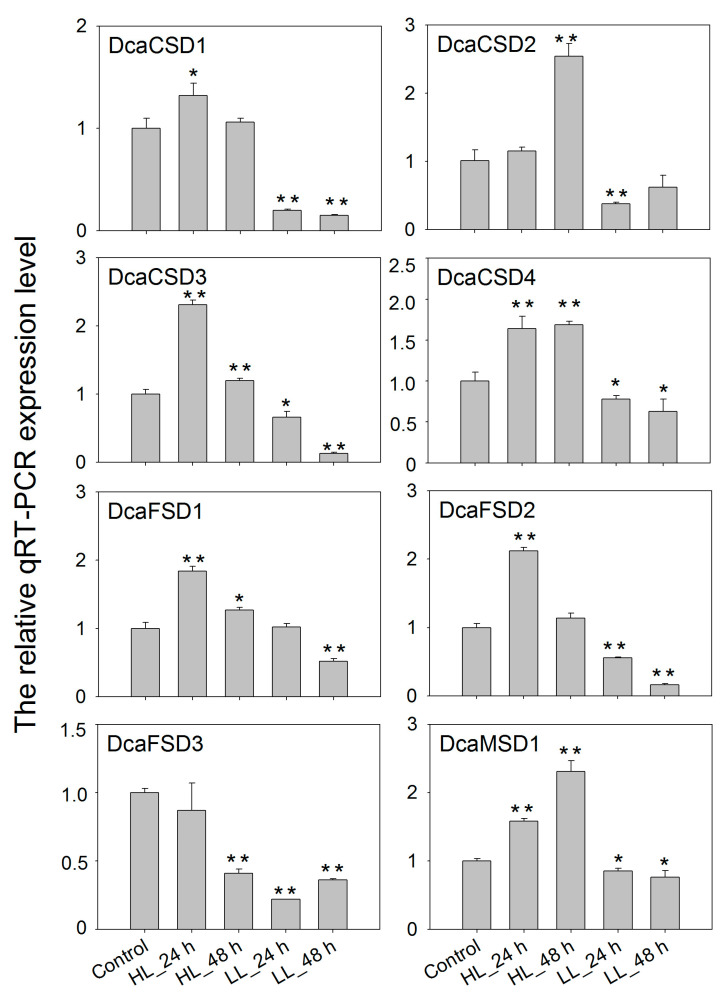
qTR-PCR analysis of the expression patterns of *DcaSOD* genes under high light and dark treatments. The relative qRT-PCR expression levels were calculated with 2^−^^∆∆CT^ and the *DcaActin* gene was used as endogenous reference gene. Error bars represent the standard deviation of three replications. Bars marked with asterisks indicate significant differences (Student’s *t*-test) to corresponding control samples for the time points under treatments (* *p* < 0.05, ** *p* < 0.01).

**Figure 8:**
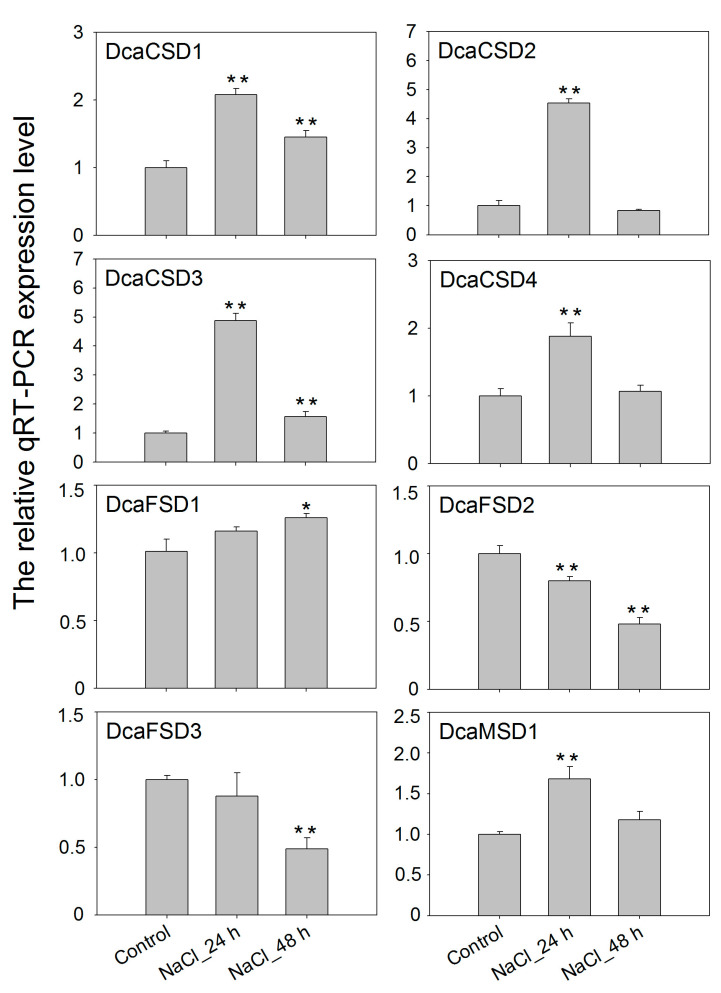
qTR-PCR analysis of the expression patterns of *DcaSOD* genes under NaCl treatment. The relative qRT-PCR expression levels were calculated with 2^−^^∆∆CT^ and the *DcaActin* gene was used as endogenous reference gene. Error bars represent the standard deviation of three replications. Bars marked with asterisks indicate significant differences (Student’s *t*-test) to corresponding control samples for the time points under treatments (* *p* < 0.05, ** *p* < 0.01).

To gain insight into potential functions, qRT-PCR was used to assess the heat (35 °C), high light (HL), dark (LL) and NaCl treatments on the expression of eight *DcaSOD* genes in *D. catenatum* (Figure 6). Under heat stress, *DcaCSD1* and *DcaCSD3* were markedly up-regulated with the extension of time, especially in 48 h. The expression levels *DcaCSD2* significantly increased in 4 h and 24 h, and followed with a decrease expression level in 48 h. The *DcaFSD1*, *DcaFSD2*, *DcaFSD3* and *DcaMSD1* were down-regulated under heat stresses, except for the *DcaFSD1* in 48 h, *DcaFSD3* in 24 h and *DcaMSD1* in 4 h (Figure 6). Expression levels of seven *DcaSOD* genes including *DcaCSD1*, *DcaCSD2*, *DcaCSD3*, *DcaCSD4, DcaFDS1*, *DcaFDS2* and *DcaMSD1*, were markedly up-regulated in leaves after HL treatments. The expression level of *DcaCSD3* under HL treatment for 24 h was two times than that of control. It is notable that LL suppressed the expression of almost *DcaSOD* genes (Figure 7). Under salt stress, six *DcaSOD* genes including *DcaCSD1*, *DcaCSD2*, *DcaCSD3*, *DcaCSD4*, *DcaFSD1,* and *DcaMSD1* were up-regulated in 24 h, especially the members of CSD subfamily. With the extension of salt stress time to 48 h, the expression levels of these *DcaSOD* genes were down-regulated compared with 24 h, except for the *DcaFSD1* (Figure 8).

## 3. Discussion

Environmental stresses pose considerable challenges for plant growth and development. SODs are the core of antioxidant enzymes, and can effectively reduce oxidative damage via scavenging the active oxygen produced by organisms under stress. The crucial roles of SOD genes in the acclimation of plants to abiotic stresses have been demonstrated in many previous studies. However, detailed information concerning *DcaSODs* characters and functions, particularly their role in stresses responses of *D. catenatum*, remained unclear. Therefore, a systematic analysis of the SOD gene family was performed in *D. catenatum*.

In this study, a total of eight *DcaSOD* genes were identified in *D. catenatum* genome. Compared with other plant species, the number of *SOD* genes in *D. catenatum* is close to *A. thaliana* (8), tomato (8), cucumber (9), and *S. miltiorrhiza* (8), which is less than that in banana (12), cotton (*Gossypium hirsutum*, 18), Wheat (26). Intragenome syntenic relationship analysis indicated that *MaCSD2A* and *2B*, and *MaMSD1A* and *1C* or *1D* in banana genome were derived from whole genome duplication [15]. Differences in the number of *SOD* genes between plant species may be attributed to gene duplication, which comprises tandem and segmental duplication, and plays a crucial role in the expansion of *SOD* genes for diversification. Analysis of the evolutionary relationships of SOD proteins among *D. catenatum, Arabidopsis*, *O. sativa*, *P. equestris* and *A. shenzhenica* showed that SOD proteins could be divided into three subfamily based on their metal co-factors, namely CSD, FSD, and MSD subfamily (Figure 1). Previous studies showed that there are only FeSOD and MnSOD in algae and bryophytes. Cu/ZnSOD only exist in higher plants, implying that FeSOD and MnSOD evolved first, and then Cu/ZnSOD appeared later to cope with the complex external environment that affects plant growth and development [22]. We found that all DcaSOD proteins were much closer to PeqSOD proteins than AshSOD proteins. There is a much closer evolutionary relationship between *D. catenatum* and *P. equestris* than *D. catenatum* and *A. shenzhenica* based on the whole genome sequences analysis [23]. The conserved motifs and gene structures provided further support for the classification of DcaSOD proteins in *D. catenatum*. The DcaFSD3 has a shorter length compared to other DcaSODs and FSDs in other species. We infer that the gene has been truncated, and key domains are missing. The failure of subcellular location prediction, and few identified motifs and exons in DcaFSD3 might attribute to its truncated sequence. Most DcaSOD proteins of the same group apparently had similar motifs constituents (Figure 2), which is in accordance with the results in other plant species [15,17,18]. Taken together, the similarities in conserved motifs and gene structures in the same subfamily corroborate their classification and inferred evolutionary relationships.

In *D. catenatum*, most *DcaSOD* genes displayed distinct tissue-specific expression patterns (Figure 5a). Most of them showed higher expression level in flower, which is similar to previous studies on foxtail millet [24] and *Zostera marina* [25]. There is a high production of ROS during organogenesis and reproductive metabolism, and high expression levels of *SOD* gene in flowers [18,19]. Moreover, most *DcaSOD* genes were highly expressed in leaf. *MaCSD1D* and *MaFSD1A* in banana exhibited the highest expression levels in leaves. However, other *MaSODs* were expressed moderately in leaves [15]. In cucumber, the expression of *CsCSD1*, *CsCSD2*, *CsFSD1*, *CsFSD2* were also abundantly expressed in leaves [18]. The preferential expression patterns of *SOD* genes imply their specific roles in the development and biological function of different tissues. In addition, there might be functional divergence of *SODs* in different species.

A lot of evidence demonstrated that *SOD* genes participated in abiotic stresses responses [12,13,14,15]. A total of 142 *cis*-elements related to hormones and stresses responses were identified in all identified *DcaSOD* promoters (Figure 4). Among these predicted *cis*-elements, the light responsiveness elements (77) were the largest group element and appeared in six *DcaSOD* promoters (Figure 4). In cucumber, the light-responsive elements were also the largest group of elements [18]. A relatively large number of light-responsive *cis*-elements was also observed in tomato *SiSOD* promoters [17]. These results might suggest that the *SOD* genes participate in light response, which was confirmed by our qRT-PCR analysis (Figure 6). Under high and low light conditions, almost *DcaSOD* genes showed different expression levels. The high light promoted the expression of all *DcaSOD* genes, except for *DcaFSD3*. Transgenci pea with an overexpressing chloroplastic Cu/ZnSOD showed increased resistant to high light [26]. Although only three low-temperature responsiveness elements were found in *DcaCSD2* and *DcaFSD1* genes (Figure 4), almost *DcaSODs* were up-regulated under cold treatment (Figure 5b), demonstrating that those *DcaSODs* might play a predominant antioxidant role under cold stress. The introduction of *MnSOD* into the mitochondria and chloroplast of alfalfa resulted in an improvement of freeze tolerance [27]. Several *cis*-elements involved in drought response including ABA responsiveness, drought-inducibility and defense and stress responsiveness were identified in *DcaCSD4*, *DcaFSD2*, *DcaFSD3* and *DcaMSD1* (Figure 4). RNA-Seq data analysis results showed that all *DcaSODs* were up-regulated under drought treatment (Figure 5). Drought tolerance of sugarcane was attributed to the elevated activity of SOD [28]. Han et al. [21] demonstrated that *SmCSD2* and *SmCSD3* were highly expressed under drought stress in *S. miltiorrhiza*. Furthermore, the Pearson correlation analysis revealed that several genes function as stress resistance had close relationship with *DcaCSD1* and *DcaCSD2*. For example, a *glutathione peroxidase* (DN33357_c2_g2_i1) that constitutes a glutathione peroxidase-like protective system against oxidative stresses [29], has a close relationship with *DcaCSD1*. The *proline-rich receptor-like protein kinase* (PERK) (DN29768_c0_g1_i3) has a close relationship with *DcaCSD2*. PERK suppressed the accumulation of ROS in *Arabidopsis* root [30]. The correlation analysis could provide useful information to reveal the regulation network of important pathways [31,32]. Under heat stress, the expression levels of four *DcaCSDs* increased at three time points, except for *DcaCSD1* and *DcaCSD4* at 4 h and *DcaCSD2* at 48 h. However, the expression patterns of three *DcaFSDs* were opposite to that of *DcaCSDs*, and deceased under heat stress at 4 h. Heat stress can cause photoinhibition of PSII and promotes the accumulation of ROS, which accelerates oxidative stress [33]. Heat stress represses the expression of chloroplastic *MaCSD2A* but strongly induces chloroplastic *MaFSD1A* in banana [15]. It must be aware that there are different mechanisms of antioxidants to response the short-time (minutes and hours) reaction and the long-term adaptation in different light and temperature treatments [34]. Under NaCl stress, four *DcaCSDs* gene and *DcaMSD1* were significantly up-regulated in 24 h, especially *DcaCSD2* and *DcaCSD3*, and then decreased in 48 h. Contrast to *DcaCSDs* and *DcaMSD1*, three *DcaFSDs* were down-regulated or unchanged. We infer that *DcaCSDs* and *DcaMSD1* genes play important role in coping with salt stress. Wang et al. [35] reported that transgenic *Arabidopsis* overexpressing *MnSOD* enhanced salt-tolerance. Further studies are needed to clarify the role of *DcaSODs* in the future.

## 4. Materials and Methods

### 4.1. Identification and Sequence Analysis of DcaSOD Genes in D. catenatum

The SOD proteins sequences of *A. thaliana* and *O. sativa* were retrieved from The Arabidopsis Information Resource (TAIR) (https://www.arabidopsis.org/) and Phytozome (http://www.phytozome.net/). The genome sequences of the *D. catenatum, P. equestris* and *A. shenzhenica* were downloaded from NCBI under the accession codes JSDN00000000 [6], PRJNA389183 and PRJNA310678 [23]. Firstly, SOD proteins sequences from *Arabidopsis* and *O. sativa* as query sequences to search the *D. catenatum, P. equestris* and *A. shenzhenica* protein database for candidate sequences by BLASTP (*E*-value < 1 × 10^−5^). In addition, then, the hmmsearch program of the HMMER software (version 3.2.1) (http://hmmer.org/download.html) was also applied to the identification of Fe/MnSOD (PF02777 and PF00081) and Cu/ZnSOD (PF00080) in Pfam 32.0 database (http://pfam.xfam.org/). For further screening, the above obtained protein sequences were analyzed by SMART (http://smart.embl-heidelberg.de/) and NCBI Conserved Domain-search (https://www.ncbi.nlm.nih.gov/cdd). Physicochemical characteristics of the DcaSOD proteins were computed using the online ExPASy-ProtParam tool (http://web.expasy.org/protparam/), including the number of amino acids, molecular weight (MW), isoelectric point (pI) and grand average of hydropathicity (GRAVY). The subcellular localization of DcaSOD proteins were predicted by ProComp 9.0 (http://linux1.softberry.com).

### 4.2. Phylogenetic Analysis

All predicted DcaSODs together with SODs of *Arabidopsis*, *O. sativa*, *P. equestris* and *A. shenzhenica* were aligned with CLUSTAL. A Maximum-Likelihood (ML) phylogenetic tree was constructed by MEGA X (version 10.1.7) [36], with the bootstrap values of 1000 replicates. The phylogenetic tree was visualized by iTOL (https://itol.embl.de/) [37].

### 4.3. Conserved Motifs, Gene Structures, Locations, Multiple Sequence Alignments and Cis-elements Analyses of DcaSOD Genes

The distribution of the conserved motifs based on amino acid sequence was conducted with the MEME (http://meme.nbcr.net/meme/) [38] and was visualized by using TBtools [39]. The exon-intron structures of *DcaSOD* genes were analyzed by the Gene Structure Display Server (GSDS) (http://gsds.cbi.pku.edu.cn/). According to the *D. catenatum* genome annotation file, the start and end location information of *DcaSOD* genes were extracted and visualized by TBtools. Multiple sequence alignments of the amino acid sequences of DcaSOD proteins were performed by ClustalW software (https://www.genome.jp/tools-bin/clustalw) and then redrawn with ESPript 3.0 (http://espript.ibcp.fr/ESPript/ESPript/index.php). For *cis*-acting regulatory elements predication, the DNA sequences (2000 bp) upstream of the initiation codon for each candidate gene were extracted, and the *cis*-elements were predicted with PlantCARE (http://bioinformatics.psb.ugent.be/webtools/plantcare/html/) [40].

### 4.4. Transcriptome Analysis

The raw RNA-seq data of different tissues under cold and dry treatments of *D. catenatum* were downloaded from the NCBI Sequence Read Archive (SRA) database (http://www.ncbi.nlm.nih.gov/sra) under the BioProject number PRJNA283237, PRJNA314400, and PRJNA432825 [41]. Hisat [42] was used for mapping reads to the *D. catenatum* reference genome with default parameters. The Stringtie [43] was used to analyze gene expression level, and then the Fragments Per Kilobase Million (FPKM) value was used to normalize gene expression level. The DEseq2 [44] was employed to identify the differentially expressed genes (DEGs) with a threshold of fold change ≥ 2 and false discovery rate (FDR) ≤ 0.01. The heatmaps of gene expression and the correlation coefficient calculation were performed by R software.

### 4.5. Plant Material, Growth Conditions and Treatments

*D. catenatum* three-year old plantlets were used in this study. For light treatments, the plantlets were treated under dark (designed as LL), high light (250 μmol photons m^−2^s^−1^, designed as HL) and 12 h light (80 μmol photons m^−2^s^−1^)/12 h dark photoperiod (control) for 24 and 48 h in greenhouse. For heat treatment, the plantlets were treated at 35 °C for 4, 24 and 48 h, and 25 °C was used as control in a growth chamber. Under salt stress, plantlets were treated with 0.5 M NaCl and sterile water (control) for 24 and 48 h in greenhouse. After treatments, mature leaves were collected and frozen immediately in liquid nitrogen, and stored at −80 °C for RNA extraction. Three biological replicate samples were contained in each treatment.

### 4.6. Real-Time PCR Experiment

Total RNAs were extracted using RNAprep Pure kit (DP441, Tiangen, Beijing, China), and were used as template to synthesize the first-strand cDNA by using the FastKing RT kit (Tiangen, Beijing, China). The primers were designed based on *DcaSOD* genes sequences using Premier 5.0 software (Table S4). The *DcaActin* gene was selected as an internal standard. qRT-PCR was performed on ABI PRISM^®^ 7500 Sequence Detection System (Applied Biosystems, Foster City, CA, USA). qRT-PCR assay was performed as described Yao et al. [45]. The 2^–ΔΔCT^ method was used to analyze relative transcript abundances. Student’s *t*-test was employed using SPSS software (version 18.0) to calculated levels of significance (* *p* < 0.05, ** *p* < 0.01).

## Figures and Tables

**Table 1:** Summary of physicochemical characteristics and classification of DcaSOD proteins in *D. catenatum*.

No.	Gene Name	Gene ID	Functional Annotations	Position	Protein Length (aa)	MW	*pI*	Predicted Subcellular Localization	Grand Average of Hydropathicity (GRAVY)
1	*DcaCSD1*	Dca002609	Cu/ZnSOD	KZ503041.1:12,499,497–2,513,682	224	22,761.91	6.43	Chloroplast	0.086
2	*DcaCSD2*	Dca003218	Cu/ZnSOD	KZ501977.1:4,636,681–4,648,211	152	15,312.98	5.34	Cytoplasmic	−0.208
3	*DcaCSD3*	Dca012338	Cu/ZnSOD	KZ503541.1:64,465–66,952	222	22,926.95	6.29	Cytoplasmic	−0.008
4	*DcaCSD4*	Dca016361	Cu/ZnSOD	KZ502953.1:468,317–473,374	164	16,547.33	6.16	Cytoplasmic	−0.151
5	*DcaFSD1*	Dca023874	FeSOD	KZ501928.1:290,962–306,815	228	26,325.25	6.71	Chloroplast	−0.344
6	*DcaFSD2*	Dca004481	FeSOD	KZ502877.1:8,227,560–8,230,822	269	30,783.39	4.91	Chloroplast	−0.488
7	*DcaFSD3*	Dca024864	FeSOD	KZ503331.1:79–387	76	26,554.21	5.66	None	0.603
8	*DcaMSD1*	Dca024548	MnSOD	KZ502716.1:147,498–155,759	238	26,554.21	8.61	Mitochondrial	−0.343

**Table 2:** The Pearson correlation coefficients between two *DcaCSD* genes and other DEGs. The FPKM values of these genes are listed in Table S3. The genes with a correlation coefficients > 0.99 with *DcaCSD1* have been indicated by red. The genes with a correlation coefficients > 0.99 with *DcaCSD2* have been indicated by blue.

Gene ID	Correlation Coefficient	*p*-Value	Description
DcaCSD1			
DN40231_c3_g1_i3	0.999	0.0004	MADS box protein DOMADS2
DN32316_c5_g1_i3	0.999	0.0005	HVA22-like protein e
DN28998_c0_g1_i2	−0.999	0.0007	Cytochrome P450 90B2
DN33200_c0_g6_i4	0.999	0.0008	/
DN37544_c1_g1_i1	0.999	0.0012	CDT1-like protein a, chloroplastic
DN31187_c4_g1_i2	0.999	0.001	Sucrose synthase
DN36361_c1_g5_i2	−0.997	0.0026	Trimethyltridecatetraene synthase
DN33357_c2_g2_i1	0.996	0.0038	Putative glutathione peroxidase 7, chloroplastic
DN24425_c0_g1_i2	0.996	0.0039	Dermcidin
DN32586_c2_g4_i1	0.996	0.0041	Aldehyde dehydrogenase family 2 member C4
DN36550_c0_g2_i4	0.996	0.0041	DEAD-box ATP-dependent RNA helicase 50
DN29711_c0_g1_i2	0.996	0.0044	Keratin, type I cytoskeletal 14
DN78496_c0_g1_i1	0.996	0.0044	Apolipoprotein E
DN39306_c2_g1_i5	0.995	0.0048	Keratin, type I cytoskeletal 10
DN29035_c0_g1_i1	0.995	0.0049	Probable histone H2A.2
DcaCSD2			
DN29768_c0_g1_i3	−0.995	0.0046	Proline-rich receptor-like protein kinase PERK13
DN33141_c1_g5_i1	−0.995	0.0049	/
DN33417_c1_g1_i2	−0.999	0.0013	Zinc-finger homeodomain protein 2
DN34829_c2_g1_i11	−0.997	0.0027	Receptor-like serine/threonine-protein kinase SD1-7
DN36012_c3_g1_i1	−0.999	0.0008	WAT1-related protein At5g64700
DN36361_c1_g1_i2	−0.996	0.0037	Trimethyltridecatetraene synthase
DN36986_c4_g1_i2	0.997	0.0033	Starch branching enzyme I
DN37712_c8_g3_i2	−0.999	0.0003	3-O-acetylpapaveroxine carboxylesterase CXE1
DN38190_c0_g2_i6	−0.997	0.0027	Ricin B-like lectin R40C1
DN38200_c5_g1_i1	−0.995	0.0049	/
DN38969_c0_g3_i6	−0.998	0.0020	RNA-directed DNA polymerase homolog
DN40032_c2_g2_i2	−0.999	0.0008	Transposon Ty3-I Gag-Pol polyprotein
DN40530_c1_g4_i2	0.998	0.0025	Probable alpha-mannosidase At5g13980
DN40586_c9_g5_i1	0.997	0.0030	Homeobox-leucine zipper protein HOX32
DN40622_c1_g3_i1	−0.998	0.0018	Protein ASPARTIC PROTEASE IN GUARD CELL 1

## Supplementary Materials

Supplementary Materials can be found at https://www.mdpi.com/2223-7747/9/11/1452/s1. Table S1: The SOD protein sequences of D. catenatum, Arabidopsis, O. sativa, P. equestris and A. shenzhenica. Table S2: The predicted cis-elements of DcaSOD genes promoters. Table S3: The annotation and expression level of DEGs under drought. Table S4: qRT-PCR primers used in this study.
