# Supplementary material for: Insights into the Superoxide Dismutase Gene Family and Its Roles in Dendrobium catenatum under Abiotic Stresses
Source: Plants (Basel). 2020 Oct 28;9(11):1452. doi: 10.3390/plants9111452 (PMC7693223; doi:10.3390/plants9111452)
Supplement: Supplementary file 1 [file plants-09-01452-s001.zip › Table 1.doc]

| No. | Gene name | Gene ID | Functional  annotations | Position | Protein length (aa) | MW | *pI* | Predicted Subcellular Localization | Grand average of hydropathicity (GRAVY) |
| --- | --- | --- | --- | --- | --- | --- | --- | --- | --- |
| 1 | *DcaCSD1* | Dca002609 | Cu/ZnSOD | KZ503041.1:12499497-12513682 | 224 | 22761.91 | 6.43 | Chloroplast | 0.086 |
| 2 | *DcaCSD2* | Dca003218 | Cu/ZnSOD | KZ501977.1:4636681**-**4648211 | 152 | 15312.98 | 5.34 | Cytoplasmic | -0.208 |
| 3 | *DcaCSD3* | Dca012338 | Cu/ZnSOD | KZ503541.1:64465**-**66952 | 222 | 22926.95 | 6.29 | Cytoplasmic | -0.008 |
| 4 | *DcaCSD4* | Dca016361 | Cu/ZnSOD | KZ502953.1:468317**-**473374 | 164 | 16547.33 | 6.16 | Cytoplasmic | -0.151 |
| 5 | *DcaFSD1* | Dca023874 | FeSOD | KZ501928.1:290962**-**306815 | 228 | 26325.25 | 6.71 | Chloroplast | -0.344 |
| 6 | *DcaFSD2* | Dca004481 | FeSOD | KZ502877.1:8227560**-**8230822 | 269 | 30783.39 | 4.91 | Chloroplast | -0.488 |
| 7 | *DcaFSD3* | Dca024864 | FeSOD | KZ503331.1:79**-**387 | 76 | 26554.21 | 5.66 | None | 0.603 |
| 8 | *DcaMSD1* | Dca024548 | MnSOD | KZ502716.1:147498**-**155759 | 238 | 26554.21 | 8.61 | Mitochondrial | -0.343 |

Table 1. Summary of physicochemical characteristics and classification of DcaSOD proteins in *D. catenatum*.
