# Supplementary material for: Insights into the Superoxide Dismutase Gene Family and Its Roles in Dendrobium catenatum under Abiotic Stresses
Source: Plants (Basel). 2020 Oct 28;9(11):1452. doi: 10.3390/plants9111452 (PMC7693223; doi:10.3390/plants9111452)
Supplement: Supplementary file 1 [file plants-09-01452-s001.zip › Table 2..doc]

Table 2. The Pearson correlation coefficients between two *DcaCSD* genes and other DEGs. The FPKM values of these genes are listed in Table S3. The genes with a correlation coefficients > 0.99 with *DcaCSD1* have been indicated by red. The genes with a correlation coefficients > 0.99 with *DcaCSD2* have been indicated by blue.

| Gene ID | Correlation Coefficient | *p*-Value | Description |
| --- | --- | --- | --- |
| DcaCSD1 |  |  |  |
| DN40231_c3_g1_i3 | 0.999 | 0.0004 | MADS box protein DOMADS2 |
| DN32316_c5_g1_i3 | 0.999 | 0.0005 | HVA22-like protein e |
| DN28998_c0_g1_i2 | -0.999 | 0.0007 | Cytochrome P450 90B2 |
| DN33200_c0_g6_i4 | 0.999 | 0.0008 | / |
| DN37544_c1_g1_i1 | 0.999 | 0.0012 | CDT1-like protein a, chloroplastic |
| DN31187_c4_g1_i2 | 0.999 | 0.001 | Sucrose synthase |
| DN36361_c1_g5_i2 | -0.997 | 0.0026 | Trimethyltridecatetraene synthase |
| DN33357_c2_g2_i1 | 0.996 | 0.0038 | Putative glutathione peroxidase 7, chloroplastic |
| DN24425_c0_g1_i2 | 0.996 | 0.0039 | Dermcidin |
| DN32586_c2_g4_i1 | 0.996 | 0.0041 | Aldehyde dehydrogenase family 2 member C4 |
| DN36550_c0_g2_i4 | 0.996 | 0.0041 | DEAD-box ATP-dependent RNA helicase 50 |
| DN29711_c0_g1_i2 | 0.996 | 0.0044 | Keratin, type I cytoskeletal 14 |
| DN78496_c0_g1_i1 | 0.996 | 0.0044 | Apolipoprotein E |
| DN39306_c2_g1_i5 | 0.995 | 0.0048 | Keratin, type I cytoskeletal 10 |
| DN29035_c0_g1_i1 | 0.995 | 0.0049 | Probable histone H2A.2 |
| DcaCSD2 |  |  |  |
| DN29768_c0_g1_i3 | -0.995 | 0.0046 | Proline-rich receptor-like protein kinase PERK13 |
| DN33141_c1_g5_i1 | -0.995 | 0.0049 | / |
| DN33417_c1_g1_i2 | -0.999 | 0.0013 | Zinc-finger homeodomain protein 2 |
| DN34829_c2_g1_i11 | -0.997 | 0.0027 | Receptor-like serine/threonine-protein kinase SD1-7 |
| DN36012_c3_g1_i1 | -0.999 | 0.0008 | WAT1-related protein At5g64700 |
| DN36361_c1_g1_i2 | -0.996 | 0.0037 | Trimethyltridecatetraene synthase |
| DN36986_c4_g1_i2 | 0.997 | 0.0033 | Starch branching enzyme I |
| DN37712_c8_g3_i2 | -0.999 | 0.0003 | 3-O-acetylpapaveroxine carboxylesterase CXE1 |
| DN38190_c0_g2_i6 | -0.997 | 0.0027 | Ricin B-like lectin R40C1 |
| DN38200_c5_g1_i1 | -0.995 | 0.0049 | / |
| DN38969_c0_g3_i6 | -0.998 | 0.0020 | RNA-directed DNA polymerase homolog |
| DN40032_c2_g2_i2 | -0.999 | 0.0008 | Transposon Ty3-I Gag-Pol polyprotein |
| DN40530_c1_g4_i2 | 0.998 | 0.0025 | Probable alpha-mannosidase At5g13980 |
| DN40586_c9_g5_i1 | 0.997 | 0.0030 | Homeobox-leucine zipper protein HOX32 |
| DN40622_c1_g3_i1 | -0.998 | 0.0018 | Protein ASPARTIC PROTEASE IN GUARD CELL 1 |
