# Supplementary material for: Insights into the Superoxide Dismutase Gene Family and Its Roles in Dendrobium catenatum under Abiotic Stresses
Source: Plants (Basel). 2020 Oct 28;9(11):1452. doi: 10.3390/plants9111452 (PMC7693223; doi:10.3390/plants9111452)
Supplement: Supplementary file 1 [file plants-09-01452-s001.zip › Table S4.doc]

Table S4. Primers used in this study

| Gene ID | Primer |
| --- | --- |
| *DcaCSD1* | F 5' AACCCAGCAGCCGTACCCAA 3' |
|  | R 5' CGACCTGAGAACTTCCCTTGA 3' |
| *DcaCSD2* | F 5' AAGAAGTGAAGGGCACAATC 3' |
|  | R 5' GAGCACCAGTGGACAAGCAG 3' |
| *DcaCSD3* | F 5' ACGGGACTTACTCCAGGATC 3' |
|  | R 5' GTGTCAGGCTGTTAGGGTTG 3' |
| *DcaCSD4* | F 5' AAGGGAGTGGTTCTTATTGC 3' |
|  | R 5' GTCACCGAAGGAGTGGATAT 3' |
| *DcaFSD1* | F 5' CCCTCCCTATAAACTAGATGC 3' |
|  | R 5' CCTCCAAGGTGTAGCCGTAT 3' |
| *DcaFSD2* | F 5' CCCCTCAGCCGAAGACAATA 3' |
|  | R 5' AACATCAATGGCAAGCAACG 3' |
| *DcaFSD3* | F 5' TTACATGATACTTCCTGCTC 3' |
|  | R 5' ATTCAAGATTGTTACCTCCC 3' |
| *DcaMSD1* | F 5' AGGGAACTGGTTTCGTGTCG 3' |
|  | R 5' AGGGCTTTGTTGTAGTTTGTGATG 3' |
| *DcaActin* | F 5' AGTTCCTATTTATGAGGGTTATGC 3' |
|  | R 5' CCTGACAATTTCACGCTCTG 3' |
